# Diabetes management in Wolcott-Rallison syndrome: analysis from the German/Austrian DPV database

**DOI:** 10.1186/s13023-020-01359-y

**Published:** 2020-04-22

**Authors:** Alena Welters, Thomas Meissner, Katja Konrad, Clemens Freiberg, Katharina Warncke, Sylvia Judmaier, Olga Kordonouri, Michael Wurm, Matthias Papsch, Gisela Fitzke, Silke Christina Schmidt, Sascha R. Tittel, Reinhard W. Holl

**Affiliations:** 1 Department of General Paediatrics, Neonatology and Paediatric Cardiology, University Children’s Hospital Düsseldorf, Düsseldorf, Germany; 2 Department of Paediatric and Adolescent Medicine, Elisabeth-Hospital Essen, Essen, Germany; 3 Department of Paediatrics, Universitätsmedizin Göttingen, Göttingen, Germany; 4 Department of Paediatrics, Kinderklinik München Schwabing, Technical University of Munich School of Medicine, Munich, Germany; 5 Department of Paediatrics, LKH Hochsteiermark/Standort Leoben, Leoben, Austria; 6 Diabetes Centre for Children and Adolescents, Kinder- und Jugendkrankenhaus Auf der Bult, Hannover, Germany; 7 Department of Paediatrics, St. Hedwigs Campus, University Children’s Hospital Regensburg, Regensburg, Germany; 8 Department of Paediatrics, Marienhospital, Gelsenkirchen, Germany; 9 Department of Paediatrics, Ostalbklinikum Aalen, Aalen, Germany; 10 Department of Paediatrics and Adolescent Medicine, Klinikum Dritter Orden, Munich, Germany; 11 Institute of Epidemiology and Medical Biometry, ZIBMT, University of Ulm, Ulm, Germany; 12 German Centre for Diabetes Research (DZD), Munich-Neuherberg, Germany

**Keywords:** *EIF2AK3* mutation, Neonatal diabetes, Wolcott-Rallison syndrome, DPV registry

## Abstract

**Background:**

Wolcott-Rallison syndrome (WRS) is characterized by permanent early-onset diabetes, skeletal dysplasia and several additional features, e.g. recurrent liver failure. This is the first multicentre approach that focuses on diabetes management in WRS. We searched the German/Austrian Diabetes-Patienten-Verlaufsdokumentation (DPV) registry and studied anthropometric characteristics, diabetes treatment, glycaemic control and occurrence of severe hypoglycaemia (SH) and diabetic ketoacidosis (DKA) in 11 patients with WRS. Furthermore, all local treatment centres were personally contacted to retrieve additional information on genetic characteristics, migration background and rate of consanguinity.

**Results:**

Data were analysed at diabetes onset and after a median follow-up period of 3 (1.5–9.0) years (time from diagnosis to latest follow-up). Median age at diabetes onset was 0.2 (0.1–0.3) years, while onset was delayed in one patient (aged 16 months). Seventy percent of patients manifested with DKA. At follow-up, 90% of patients were on insulin pump therapy requiring 0.7 [0.5–1.0] IU of insulin/kg/d. More than two third of patients had HbA1c level ≥ 8%, 40% experienced at least one episode of SH in the course of the disease. Three patients died at 0.6, 5 and 9 years of age, respectively. To the best of our knowledge three patients carried novel mutations in *EIF2AK3*.

**Conclusion:**

Insulin requirements of individuals with WRS registered in DPV appear to be comparable to those of preschool children with well-controlled type 1 diabetes, while glycaemic control tends to be worse and episodes of SH tend to be more common. The majority of individuals with WRS in the DPV registry does not reach glycaemic target for HbA1c as defined for preschool children (< 7.5%). International multicentre studies are required to further improve our knowledge on the care of children with WRS.

## Background

Wolcott-Rallison syndrome (WRS) is a rare autosomal recessive disorder caused by loss-of-function mutations in the *eukaryotic translation initiation factor 2α kinase 3* (*EIF2AK3*) gene encoding pancreatic PKR-like endoplasmic reticulum kinase (PERK) that phosphorylates the alpha subunit of the eukaryotic translation-initiation factor 2 (eIF2-alpha) [1]. Its cardinal clinical manifestations as initially described by CD Wolcott and ML Rallison include non-autoimmune permanent early-onset diabetes mellitus and multiple epiphyseal dysplasia [2]. Several additional features have been described, whose presence and severity vary between patients. These may be present upon diagnosis or develop later during the course of the disease and include recurrent episodes of hepatic failure (in up to 85% of patients [3]), impaired renal function, exocrine pancreatic insufficiency, osteopenia, growth retardation, anaemia, neutropenia associated with recurrent infections, hypothyroidism during stress conditions (referred to as the euthyroid sick syndrome) and developmental delay [4, 5]. However, clinical appearance highly varies among patients, even in those sharing the same mutation [3, 4, 6–9]. Although a rare disease, WRS is the most common cause of permanent neonatal diabetes mellitus (PNDM) in consanguineous families where it accounts for up to 25% of cases [4, 9, 10]. Diabetes typically manifests within the first 6 months of life but delayed onset up to 30 months of age has also been reported [3, 6]. Acute episodes of diabetic ketoacidosis (DKA) are common [11–13]. Given the extensive phenotypic variability, it has been proposed to consider WRS in any infant with permanent early-onset diabetes mellitus born from consanguineous parents or originating from isolated populations or countries in which inbreeding is frequent [4].

Diabetes in WRS is not autoimmune, therefore type 1 diabetes specific islet autoantibodies are absent [11]. Instead, studies on tissue-specific knockout and PERK-deficient cell-lines revealed that PERK is required for normal fetal and early neonatal beta cell proliferation, differentiation and beta cell function [14]. Consistently, the pancreas of patients with WRS is hypoplastic, Langerhans islets are smaller and insulin-secreting beta cells are markedly reduced within the islets [11].

Liver disease is a common manifestation of WRS, present in up to 85% of individuals with WRS, and associated with high mortality rates [3]. The majority of individuals with WRS experience intermittent episodes of acute liver failure with or without cholestasis, typically triggered by stress such as intercurrent disease or hypoglycaemia related to tight diabetes control. Likewise, management of WRS is often complicated by recurrent episodes of hypoglycaemia secondary to liver dysfunction with impaired hepatic gluconeogenesis [3, 11, 12]. In individuals with WRS, optimal diabetes management is therefore of particular importance to prevent diabetic ketoacidosis as well as episodes of hypoglycaemia with possible subsequent liver failure. The aim of this study was to characterize the management of diabetes in patients diagnosed with WRS. We searched the German/Austrian DPV (Diabetes-Patienten-Verlaufsdokumentation) database and studied the course of diabetes in 11 patients diagnosed with WRS. Additional information was retrieved by personally contacting all local treatment centres. Data were compared at diabetes onset and after a median follow-up period of 3 [1.5–9] years (median [Q1-Q3]) (time from diagnosis to latest follow-up). Thus, we provide objective information on anthropometric, clinical and genetic characteristics of individuals with WRS as well as their diabetes treatment and glycaemic control including occurrence of DKA and severe hypoglycaemia (SH).

## Results

### Patient characteristics

Eleven patients with the diagnosis of WRS are currently registered in the German/Austrian DPV database. Of these, three were male (27%) and all patients for whom this information was available had a migration background (100%, *n* = 10). One Austrian and one German patient of Kosovo-origin have previously been described (patient 7 and patient 9) [4, 15]. Median age at diabetes onset was 0.2 (0.1–0.3) years (*n* = 11). At the most recent treatment year (follow-up data), patients were 2 (1.8–12.0) years old, with a median duration of diabetes of 3 (1.5–9.0) years (n = 11). BMI-SDS was − 0.25 (− 1.1 − + 0.9, *n* = 9). Three patients died at 0.6, 5 and 9 years, respectively (Table 1).

**Table 1:** Anthropometric and clinical characteristics of individuals with WRS

	n data available	median [Q1-Q3] or %
Male	11	27
Migrational background	10	100
Age at diabetes-onset (y)	11	0.2 [0.1–0.3]
Time from diagnosis to latest follow-up (y)	11	3 [1.5–9]
Age at follow-up (y)	11	2 [1.8–12]
Events of death	11	27

### Diabetes management, glycaemic control and diabetes-related complications

Data on diabetes management, glycaemic control and diabetes-related complications are summarized in Table 2**.** All patients were treated with insulin. Data on diabetes management (insulin dose) and glycaemic control (HbA1c level) were available for six and five patients from diabetes onset, and for all patients from the most recent treatment year (follow-up).

**Table 2:** Diabetes management and diabetes-related complications in individuals with WRS

	n data available	median [Q1-Q3] or %
Manifestation with diabetic ketoacidosis	10	70
Insulin dose at manifestation (IU/kg/d)	6	0.7 [0.5–0.9]
Insulin dose at follow-up (IU/kg/d)	11	0.7 [0.5–1]
HbA1c at manifestation (%)	5	7 [5.8–10.9]
HbA1c at follow-up (%)	11	8 [7–9]
≥ 1 episodes of SH during follow-up	10	40
≥ 1 episodes of DKA during follow-up	10	10

At the most recent treatment year, the vast majority of patients were treated with CSII (90%). One patient was treated with multiple daily insulin injections at 4 injection time-points per day. The total daily insulin dose of individuals with WRS registered in DPV was 3.5 (3–4.3) IU at diabetes onset (*n* = 4) and 9.6 (6.8–12.6) IU at follow-up (*n* = 10). Insulin dose per kg body weight was 0.7 (0.5–0.9) IU at diabetes onset (*n* = 6) and 0.7 (0.5–1) IU at follow-up (*n* = 11).

Most patients had an increased HbA1c level at diabetes onset (7 [5.8–10.9] %; *n* = 5). As diabetes progressed, glycaemic control further deteriorated and HbA1c reached a level of 8.0 (7.8-9) % at follow-up (n = 11).

Data on the presence of DKA at diabetes manifestation were available for 10 patients. Of these, seven patients presented with DKA (70%). One patient experienced one further episode of DKA during follow-up. 40% of the patients experienced at least one episode of severe hypoglycaemia within the course of the disease.

### Genetic characteristics and migration background

Data on the underlying genetic mutation were available for 9 patients (Table 3). Of these, six patients carried a previously described mutation in *EIF2AK3*: the homozygous c.2707C > T, p.Arg903* mutation was detected in five individuals, of which two were siblings. All patients carrying this mutation were of Albanian- or Kosovo-origin and born to un-related parents. Two of these patients have previously been described [4, 15]. Notably, in patient 9 the nomenclature differs by one amino acid (c.2704C > T, p.Arg902*) because a different reference sequence for the mutation was applied [4]. The c.997C > T, p.Gln333* mutation was detected in one German patient of Iraqi-origin born to consanguineous parents. To the best of our knowledge three patients carried a novel mutation in *EIF2AK3*: the compound heterozygous mutation c.1474 C > T, p.(Arg492*);  c.2081 C > G, p.(Ser694*) was detected in a German female with WRS of Syrian-origin, the homozygous mutation c.1A > G, p.Met? was detected in a female with WRS of Afghan-origin and the c.568_575dupGATGATGT, p.Val193Metfs10* mutation was detected in a German female with WRS of Russian-origin. All except one patient carried a homozygous mutation in *EIF2AK3*. The only patient that carried a compound heterozygous mutation was a German female of consanguineous Syrian parents. Information on the migration background was available for 10 patients (Table 3). Of these, all patients had a migration background: two patients had immigrated themselves and 8 patients had parents that had immigrated. The majority of patients originated from regions with a high level of inbreeding (Turkey, Syria, Afghanistan, Iraq) or isolated populations (Kosovo, Albania) [16, 17]. One patient was of Russian-descent.

**Table 3:** Clinical and genetic characteristics of each individual patient with WRS

	Data at diabetes onset					Data at patient’s last clinic visit				Complications		Genetic characteristics		
	Sex	Age (y)	Ins (IU/kg/d)	HbA1c (%)	DKA	Age (y)	BMI-SDS	Ins (IU/kg/d)	HbA1c (%)	SH	DKA	Mutation in ***EIF2AK3***	Migrational background	CSG
**P 1**	f	0	n.a.	n.a.	n.a.	4	1	0.5	8	n.a.	n.a.	n.a.	n.a.	n.a.
**P 2**	f	0.1	0.5	7	yes	died (0.6)	n.a.	0.5	7	1	none	n.a.	parents: Turkey; patient: born in GER	n.a.
**P 3**	f	0.1	0.7	n.a.	yes	1.8	0.75	0.5	9.5	none	none	compound heterozygous c.1474 C > T, p.Arg492*; c.2081 C > G, p.Ser694*	parents: Syria; patient: born in GER	yes
**P 4**	f	0.3	n.a.	n.a.	yes	died (9)	−1.75	1.5	9	1	1	homozygous c.1A > G, p.Met?	parents: Afghanistan; patient: born in Iran	unclear^†^
**P 5**	f	0.2	0.7	9	yes	died (5)	−1.25	0.9	8	7	none	homozygous c.568_575dupGATGATGT, p.Val193Metfs10*	parents: Russia; patient: born in GER	no
**P 6**	m	0.2	1.1	6	yes	2	1.25	0.7	7	none	none	homozygous c.997C > T, p.Gln333*	parents: Iraq; patient: born in GER	yes
**P 7**	m	0.2	0.5	5.6	no	15.5	n.a.	0.7	8.6	none	none	homozygous c.2707C > T, p.Arg903*	parents: Kosovo; patient: born in Austria	no
**P 8**	f	0.3	0.79	12.7	yes	1	−0.75	0.7	8	none	none	homozygous p.Arg903*	parents: Albania; patient: born in GER	no
**P 9**	m	0.3	n.a.	n.a.	yes	12	−1	1.1	9	none	none	homozygous c.2707C > T, p.Arg902*	parents: Kosovo; patient: born in GER	no
P 10*	f	0.1	n.a.	n.a.	no	2	0.25	1	8	4	none	homozygous c.2707C > T, p.Arg903*	parents: Kosovo; patient: born in GER	no
P 11*	f	1.3	n.a.	n.a.	no	5	−0.25	0.9	7	none	none	homozygous c.2707C > T, p.Arg903*	parents: Kosovo; patient: born in Kosovo	no

CSG: consanguinity, DKA: diabetic ketoacidosis, GER: Germany, Ins: insulin dose, n.a.: data not available, SH: severe hypoglycaemia. *these patients are siblings. ^†^great-grandparents related, status in parents unclear

## Discussion

This is the first study to focus on diabetes management in WRS. We studied anthropometric characteristics, diabetes treatment, glycaemic control, diabetes-related complications (SH and DKA) and genetic characteristics in 11 patients with WRS registered in the German/Austrian DPV registry.

The key presenting feature of WRS is PNDM. In Europe and within the DPV registry, the most common causes of PNMD are activating mutations in *ABCC8* or *KCNJ11* encoding one of the subunits of the ATP-sensitive potassium channel (K_ATP_) [10, 18]. However, in populations in which inbreeding is frequent, e.g. in North Africa, Middle East and South Asia, K_ATP_ channel mutations are less common [5]. Instead, in consanguineous populations, *EIF2AK3* mutations causing WRS account for up to 25% of PNDM cases [5, 9, 10]. Consistently, in the DPV cohort all patients for whom this information was available had a migration background, meaning that the patients themselves had immigrated (*n* = 2) or their parents were born outside the DPV countries (*n* = 8). All except one patient originated from regions with a high level of inbreeding (Turkey, Syria, Afghanistan, Iraq) or rather small ethnic groups (Kosovo, Albania) [16, 17]. However, the rate of self-reported parental consanguinity was low (n = 2). All except one patient for whom information on the underlying mutation was available carried a homozygous mutation in *EIF2AK3*. The only patient that carried a compound heterozygous mutation was a German girl of consanguineous Syrian parents. Five patients, of which two were siblings, shared the same mutation in *EIF2AK3* (c.2707C > T, p.Arg903*). All of these patients were of Albanian- or Kosovo-origin and born to un-related parents. This mutation has previously been described in at least three additional individuals with WRS of Albanian-origin, further supporting the assumption of a founder effect in this population [17, 19, 20]. One German male individual with WRS of Iraqi-origin carried the homozygous mutation c.997C > T, p.Gln333* in *EIF2AK3.* This mutation has previously been described in three patients of Iranian-, Turkish- and Kurdish-origin, respectively [9, 21, 22].

In most children with WRS, diabetes manifests within the first 6 months of life, typically at around two to 3 months of age, but delayed onset at 14, 18, 24 and even 30 months has also been reported [3, 4, 6, 23]. Similarly, in this cohort all except one patient presented with diabetes before 4 months of age. Median age at diabetes onset was 0.2 (0.1–0.3) years. One patient was diagnosed at the age of 16 months and thus expands the number of individuals with WRS with delayed onset.

The majority of individuals with WRS in the DPV registry were on insulin pump therapy (90%), which is the preferred mode of insulin delivery for paediatric patients with type 1 diabetes aged < 7 years [24]. Daily insulin dose per kg bodyweight (IU/kg/d) in this cohort of individuals with WRS was 0.7 (0.5–0.9) IU at diabetes onset and 0.7 (0.5–1) IU at follow-up in patients aged 2 (1.8–12) years. Thus, insulin requirements of individuals with WRS appear to be comparable to that of preschool children with well-controlled type 1 diabetes mellitus (T1DM), requiring insulin doses of approximately 0.6 IU/kg/d [24], albeit the number of patients with WRS studied is too small to statistically compare these two groups.

Individuals with WRS tend to develop recurrent episodes of DKA and hypoglycaemia [3, 11–13]. In the DPV registry, seven patients presented with DKA (70%). Notably, patient 10 was closely monitored to prevent DKA, as her sister (patient 11) had been diagnosed with WRS. These observations are in line with previous reports. It has been reported that up to 65% of patients with any type of monogenic diabetes present with DKA [25]. Similarly, in other cohorts, 50–70% of individuals with WRS presented with DKA [3, 5]. 40% of individuals with WRS in the DPV registry experienced at least one episode of SH during follow-up and 20% of individuals with WRS experienced an episode of SH within the past treatment year. Thus, the frequency of SH tends to be higher in individuals with WRS compared to individuals with type 1 diabetes: 1.9% and 2.8% of children with T1DM aged < 6 years registered in DPV and the T1D Exchange clinic registry, a multisite registry that includes T1DM patients at all ages in the USA, respectively, experienced ≥ episode of SH within the past treatment year [26]. Preclinical studies revealed that in mice with a homozygous mutation of *eIF2-alpha* hypoglycaemic episodes are associated with defective hepatic gluconeogenesis [27]. Likewise, it has been hypothesized that in human individuals with WRS, mutant for the eIF2-alpha kinase PERK, liver dysfunction adds to the risk of developing hypoglycaemic episodes [11]. Therefore, regular daytime feeds may assist in preventing severe hypoglycaemia [28]. Of note, in WRS hypoglycaemia can trigger acute hepatic failure, and liver disease in WRS carries high mortality [3].

This study further revealed that less than one third of individuals with WRS in the DPV registry reached glycaemic targets for HbA1c as defined by the International Society for Pediatric and Adolescent Diabetes (ISPAD) and the American Diabetes Association (ADA) for preschool children (HbA1c < 7.5%; *n* = 3) [24, 29]. Mean HbA1c was 8.0 (7.8-9) % in individuals with WRS aged 2 (1.8-12) years and thus tends to be higher than in individuals with T1DM aged < 6 years from the DPV registry (HbA1c of 7.1% in users of continuous glucose monitoring [CGM] and 7.4% in non-CGM users) [30]. More than two thirds of individuals with WRS had HbA1c level ≥ 8% (*n* = 8). Higher HbA1c level may generally be tolerated rather than targeting a very tight control of blood glucose, as hypoglycaemia can trigger acute liver failure. Today, prognosis of WRS is poor and most patients die at a young age before long-term complications of diabetes become clinically evident [11, 31]. However, earlier recognition of WRS and increased awareness of additional features, particularly liver failure, may improve the care of children with WRS and lead to prolonged survival. Given their relatively poor metabolic control and young age at diabetes onset, these patients may have a high risk of developing long-term complications of diabetes [31].

Three of the individuals with WRS registered in DPV have died. One 7-months-old girl died of multiple organ failure following progressive kidney and liver dysfunction. One 5-year old girl died at her home, which is why the cause of death remains unclear. In the months before her death, she frequently suffered from severe metabolic derangement with simultaneous electrolyte imbalances that were increasingly difficult to manage. Presumably, the patient died in such a crisis. Notably, one 9-year old girl died from cerebral oedema following an elective surgical intervention for craniocervical instability without additional signs of organ failure. Therefore, as recommended by others, in individuals with WRS general anaesthesia should be planned with caution and closely monitored to prevent acute aggravation of the disease [32].

This study has several limitations. Although it is one of the largest studies to date that focuses on diabetes management and outcomes of diabetes care in individuals with WRS, the number of patients with WRS studied is too small to statistically compare diabetes management in WRS and type 1 diabetes, particularly when adjusting for confounding factors. This is why we did not statistically compare these two groups. However, a comparison with the treatment of other forms of diabetes must be attempted, as it has important implications for the treatment of diabetes in individuals with WRS. Therefore, we describe and discuss our observations in the context of type 1 diabetes treatment. Certainly, additional studies are required to further expand the knowledge of diabetes management in WRS to improve the care and long-term outcome of individuals with this disorder.

Since the DPV registry primarily records data on diabetes management and diabetes-related comorbidities, data on clinical features of WRS other than diabetes, genetic characteristics and migration background were scarce or not available. Efforts have been made to obtain additional information through personally contacting each treatment centre (Table 3). However, as this is a retrospective and multicentre database analysis, clinical manifestations of WRS other than diabetes could not be studied in detail.

## Conclusions

Wolcott-Rallison syndrome is a rare disorder and only little is known about the management of diabetes and glycaemic control in individuals with WRS. In WRS, diabetes manifests early in life, often with DKA. In the DPV cohort all patients for whom this information was available had a migration background and the majority of patients originated from countries with a high level of inbreeding or rather small ethnic groups. Insulin requirements of individuals with WRS appear to be comparable to preschool children with well-controlled T1DM. Glycaemic control as measured by HbA1c tends to be worse than in T1DM patients aged < 6 years registered in DPV. In fact, most of the individuals with WRS did not reach glycaemic target for HbA1c (< 7.5%), while almost half of the patients experienced at least one episode of severe hypoglycaemia (40%). We conclude that in WRS, diabetes needs intensive treatment, should be well educated and closely monitored to achieve optimal glycaemic control in order to minimize the risk of DKA and SH and to prevent long-term complications of diabetes. Beyond appropriate education, psychological support should be offered to parents of individuals with WRS, as insulin therapy in infants as such is a challenge [33]. Additional studies are required to further improve the care of children with WRS.

## Materials and methods

### DPV registry

The DPV registry is a nationwide prospective multicentre initiative that records demographic and anthropometric characteristics as well as data on diabetes management and diabetes-related comorbidities of children and adults with any type of diabetes. Currently, 485 centres in Germany, Austria, Switzerland and Luxembourg participate in the DPV initiative. Twice a year, each centre transmits its data in an anonymous form to the University of Ulm, Germany for central data aggregation and analysis. Inconsistent data are reported back to centres for verification or correction. Until September 2019, 564.734 patients with diabetes were registered in the electronic computer based documentation software DPV. Data collection and the analysis of anonymized data from the DPV registry are approved by the ethics committee of the University of Ulm, Germany and by local review boards of the participating centres.

For the present analysis we included all subjects with the clinical diagnosis of WRS recorded in the DPV registry. For each patient, data at diabetes onset and from the most recent treatment year were extracted and analysed. Local treatment centres were furthermore contacted to obtain additional information on diabetes management, genetic characteristics and migration background. The study sample encompassed 11 patients with the diagnosis of WRS from 9 different centres in Germany (*n* = 8) and Austria (*n* = 1).

### Data analysis and statistics

Anthropometric and clinical data analysed include sex, age at diabetes onset and at the most recent visit (follow-up), migration background (at least one parent or patient born outside the DPV countries), body mass index SD score (BMI-SDS) at follow-up, glycated haemoglobin A1c (HbA1c) level at diabetes onset and at follow-up (%), diabetes duration, type of insulin treatment, daily insulin dose, presence of DKA at diabetes onset and of DKA and SH at follow-up. SH was defined according to the ISPAD guidelines, i.e. an event associated with seizure, convulsion or loss of consciousness requiring the assistance of another person. DKA was defined as venous pH < 7.3 and/or serum bicarbonate < 15 mmol/l [34].

HbA1c level from different centres were mathematically standardized to the reference range of the Diabetes Control and Complication Trial (DCCT) (4.05–6.05%). BMI was calculated as weight in kg divided by the square of the height in meters (kg/m^2^). The German Health Interview and Examination Survey for Children and Adolescents (KiGGS study) was used as national reference data to derive BMI-SDS values [35].

Insulin treatment was categorized as multiple daily insulin injections or continuous subcutaneous insulin infusion (CSII). Insulin dose is expressed as total daily insulin dose (IU/d) and daily insulin dose per kilogram (kg) body weight (IU/kg/d).

Descriptive statistics are given as median (Q1-Q3) or as percentage.

## Data Availability

Data management of the DPV registry is coordinated by the Institute of Epidemiology and Medical Biometry at Ulm University, Germany based on rules consented by the participating institutions. Unaggregated patient-level data cannot be made publicly available to protect patient privacy. Interested research groups may apply for access and permission to analyse data from the DPV registry within the legal and ethical framework, to be evaluated by the external DPV science board. Consent rules and contact information are available at www.d-p-v.eu. Applications should be directed to the principal investigator of the DPV initiative reinhard.holl@uni-ulm.de.

## Abbreviations

BMI: Body mass index
CSII: Continuous subcutaneous insulin infusion
DCCT: Diabetes control and complication trial
DKA: Diabetic ketoacidosis
DPV: Diabetes-Patienten-Verlaufsdokumentation
*EIF2AK3*: *Eukaryotic translation initiation factor 2α kinase 3*
HbA1c: Glycated haemoglobin A1c
IU: International unit
kg: Kilogram
KiGGS: German health interview and examination survey for children and adolescents
PERK: Pancreatic PKR-like endoplasmic reticulum kinase
PNDM: Permanent neonatal diabetes mellitus
SH: Severe hypoglycaemia
T1DM: Type 1 diabetes mellitus
WRS: Wolcott-Rallison syndrome
